# Recognizing the Role of Insulin Resistance in Polycystic Ovary Syndrome: A Paradigm Shift from a Glucose-Centric Approach to an Insulin-Centric Model

**DOI:** 10.3390/jcm14124021

**Published:** 2025-06-06

**Authors:** Jim Parker, Lara Briden, Felice L. Gersh

**Affiliations:** 1 School of Medicine, University of Wollongong, Wollongong 2522, Australia; 2 Centre for Menstrual Cycle and Ovulation Research, University of British Columbia, Vancouver, BC V5Z 1M9, Canada; lara@larabriden.com; 3 College of Medicine, University of Arizona, Tucson, AZ 85004, USA; felicelgersh@yahoo.com

**Keywords:** polycystic ovary syndrome, insulin, insulin resistance, hyperinsulinemia, metabolic, hyperandrogenism, chronic inflammation, glucose-centric, insulin-centric

## Abstract

Polycystic ovary syndrome (PCOS) is a common metabolic–endocrine disorder affecting women of reproductive age, and insulin resistance (IR) is a key pathophysiological feature. Current medical education and clinical practice emphasize glucose-centric approaches in PCOS management, and IR testing is often overlooked due to limited emphasis in current clinical guidelines or the lack of standardized protocols. Additionally, the glucose-focused paradigm has been the standard of care for decades. However, this approach has led to delayed diagnosis of progressive metabolic and reproductive consequences, leaving many patients underdiagnosed and undertreated. Therefore, we propose a paradigm shift towards an insulin-centric model for PCOS management. This new approach aims to diagnose IR at an earlier stage, enabling the timely implementation of effective lifestyle and treatment strategies. By focusing on IR, clinicians can potentially limit the progression of PCOS-related reproductive and metabolic diseases. The insulin-centric model is a novel approach that involves comprehensive IR screening, dynamic insulin testing, personalized lifestyle and insulin-sensitizing interventions, and regular monitoring of insulin and glycemic parameters. This model could improve patient outcomes by facilitating early diagnosis of metabolic dysfunction and reducing the incidence of subsequent chronic disease. Furthermore, this model has broader implications, potentially transforming treatment approaches for various chronic diseases beyond PCOS.

## 1. Introduction

Polycystic ovary syndrome (PCOS) affects 8–13% of women and usually presents in adolescence with a complex mixture of symptoms that result from underlying metabolic and endocrine disturbance of homeostatic networks [1,2,3]. Developmental programming of inherited gene variants predisposes women with PCOS to reduced insulin sensitivity, which provided an adaptive survival advantage in ancestral environments [4,5,6,7,8]. However, in the modern environment, reduced insulin sensitivity predisposes to maladaptive metabolic, hormonal, and symptom responses [1,2,4,5,6,7,8,9,10,11]. In its early stages, PCOS can therefore be considered a reversible disturbance of physiology in response to environmental stressors, rather than a true disease entity. This characterization of PCOS is supported by the International Guidelines, which provide evidence that many of the symptoms and features of PCOS are reversible following diet, exercise, and other lifestyle interventions [12].

PCOS is associated with a number of chronic diseases and complications [1,12,13]. These include reproductive problems (subfertility, implantation failure, and miscarriage), pregnancy complications (pre-eclampsia, pre-term labor, fetal growth restriction, gestational diabetes, and stillbirth), and metabolic diseases (obesity, type 2 diabetes, metabolic syndrome, metabolic-associated liver disease, fatty pancreas disease, dyslipidemia, hypertension, renal and cardiovascular disease, and cancer) [14,15,16,17]. In many cases, the pathophysiological effects of insulin resistance (IR) and hyperinsulinemia on the ovary, brain, vascular endothelium, endometrium, placenta, and endocrine and metabolic systems are already established [17]. As a result, IR and hyperinsulinemia are now recognized to be significant contributors to these long-term health complications [15], in addition to being major drivers of the core features of PCOS, which include hypothalamic and ovulatory dysfunction, and hyperandrogenism [18,19,20,21,22].

**Figure 1:**
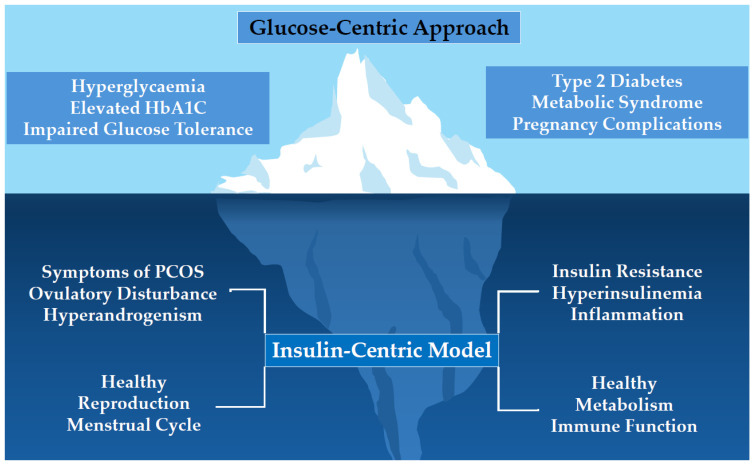
The observed features of the glucose-centric approach represent the late stages of the effects of insulin resistance or ‘’tip of the iceberg”. HbA1C = Hemoglobin A1C.

A major limitation of the prevailing glucose-centric approach is that measurable changes in serum glucose do not occur until decades after the onset of IR and hyperinsulinemia (Figure 1) [19]. As a result, IR frequently goes undetected during its early, silent phase, when intervention would be most effective [23]. In this paper, we present evidence to support a paradigm shift towards an insulin-centric model for the assessment and management of PCOS. We propose that early identification of IR in adolescents and women with PCOS would enable timely intervention and reduce the risk of subsequent metabolic and reproductive complications. This model is evidence-based, feasible, and suitable for integration into routine clinical practice. Further research is needed to improve the predictive accuracy of surrogate markers of IR, refine early intervention strategies, and enhance knowledge translation to both health professionals and women.

## 2. Scope and Methodology

The aim of this paper is to present a review of the current state of research on the advantages and disadvantages of adopting an insulin-centric model of PCOS, compared with the longstanding glucose-centric approach. Section 3, Section 4, Section 5, Section 6 and Section 7 provide the background and rationale for adopting a new approach to early diagnosis and management of metabolic disturbance in women with PCOS. This model emphasizes the central role of hyperinsulinemia and IR as driving forces that shape disease progression.

Section 3 provides an overview of the glucose-centric paradigm currently used for the assessment and management of women with PCOS. This section describes the history, rationale, testing, predictive value for detecting complications, treatment strategies, and strengths and limitations of this paradigm in a contemporary environment. Section 4 is an up-to-date summary of the physiological actions of insulin. Section 5 discusses the adaptive significance of reduced insulin sensitivity and IR in women with PCOS. Section 6 discusses the pathophysiology of IR and hyperinsulinemia in PCOS. Section 7 describes the features of the proposed insulin-centric model. This section provides evidence supporting an insulin-centric model in women with PCOS. It includes the rationale for the need to change, recommendations for testing, a plan for phase-based therapeutic interventions, and identification of future research and treatment candidates. Section 8 is the framework for an insulin-centric model and was prepared with the assistance of the Generative Artificial Intelligence (AI) tool “Microsoft Copilot”. Copilot was asked the question, “Design an insulin-centric model for the assessment and management of PCOS”.

The list of bibliographic references is based on PubMed, MEDLINE, Scopus, and Google Scholar databases. Databases were searched from inception to April 2025 repeatedly over many years. This narrative review summarizes the relevant literature and provides a new perspective on the need for greater emphasis on hyperinsulinemia and IR in women with PCOS. We propose a paradigm shift towards an insulin-centric model for the assessment and management of women with PCOS.

## 3. The Glucose-Centric Model of Insulin Resistance in PCOS

### 3.1. Origins of the Glucose-Centric Model

**Figure 2:**
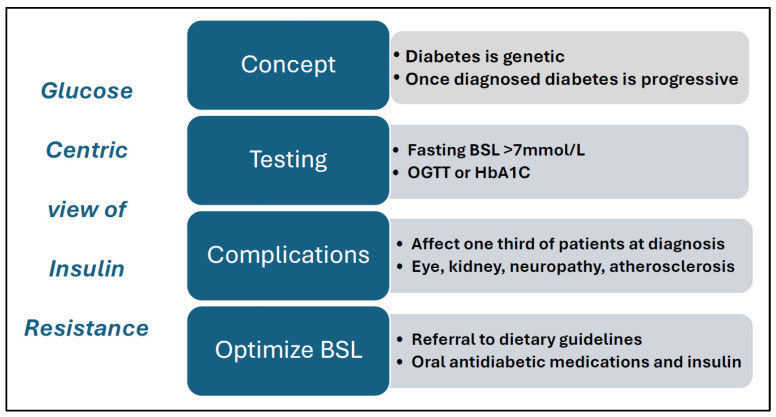
Features of a glucose-centric model of insulin resistance. Abbreviations: BSL = blood sugar levels; mmol/L = millimole per liter; OGTT = oral glucose tolerance test; HbA1C = Hemoglobin A1C.

PCOS can be a progressive metabolic disease, and women with PCOS have a significantly increased risk of developing impaired glucose tolerance (IGT), gestational diabetes mellitis (GDM), and type 2 diabetes mellitis (T2DM) [1,15,24,25,26]. As a result, the glucose-centric model of diabetes has been used for the assessment and management of PCOS (Figure 2).

Diabetes has been known since antiquity, and the term mellitis was added in the seventeenth century to describe the associated sweet taste of urine that was subsequently determined to be sugar [27]. Diabetes was also known to be associated with dietary foods such as rice, cereals, and sweets [27]. Insulin was isolated from the pancreas in 1921 and subsequently became a life-saving treatment for type 1 diabetes [28]. In 1935, Himsworth described the difference between type 1 diabetes (insulin sensitive) and T2DM (insulin insensitive) [29]. Although vaguely defined, the term IR was subsequently applied to people with insulin-insensitive T2DM [30,31].

The original description of PCOS by Stein and Levinthal emphasized the clinical features of menstrual irregularity and infertility [32]. PCOS was subsequently characterized as a reproductive disorder until it was recognized that some women with typical PCOS had acanthosis nigricans and IR [33]. Further research showed that obese women with PCOS had elevated blood glucose levels following oral glucose tolerance testing (OGTT). Large cross-sectional studies during the 1990s revealed an increased prevalence of IGT and T2DM in women with PCOS [34,35]. The current glucose-centric model of PCOS originated from the long-standing medical framework used to diagnose and manage type 1 and T2DM, for which blood glucose levels are a well-established and clinically accessible biomarker. From the point at which PCOS was recognized to be linked with metabolic dysfunction and increased risk of diabetes in the 1990s [33], the default approach has been to use glucose-based assessments such as the OGTT. Accumulating evidence now shows that IR and hyperinsulinemia precede hyperglycemia by decades and play a key role in the pathophysiology of PCOS and related complications [2,19,36,37].

### 3.2. Assessment Based on the Glucose-Centric Model of Insulin Resistance in PCOS

Many of the diabetes testing methods introduced during the twentieth century were adopted pragmatically and without robust evidence to support their accuracy or predictive value. The first clinical tests for the diagnosis of diabetes were qualitative tests to detect glucose in the urine [38]. The OGTT was introduced in 1922, and urine test strips in the 1950s (dextrostix) [39]. Glucose monitors were used in the 1970s, despite poor precision and accuracy [40]. The Hemoglobin A1c (HbA1c) test was introduced in the 1970s and provided an estimate of the average blood sugar over months rather than a single point in time [41]. In the 1980s, self-monitoring of blood glucose became the standard of care. The first continuous glucose monitor (CGM) was approved in 1999 and has been refined and evaluated over the past 25 years [42].

Tests used in the glucose-centric model in women with PCOS include fasting plasma glucose, the OGTT, and HbA1c. These tests aim to identify IGT or T2DM, rather than IR and hyperinsulinaemia. Their continued use is due in part to their standardization, accessibility, and clear diagnostic cutoffs. Nevertheless, there are many limitations to the accuracy, reproducibility, and predictive value of all these testing methods [43,44,45].

The International Guidelines for the assessment and management of PCOS advise screening for glycaemic status in all adults and adolescents with PCOS [12]. The 75 gm OGTT is recommended as a first-line test, regardless of the body mass index (BMI), as it is believed to be the most accurate. According to the Guidelines, “If an OGTT cannot be performed, fasting plasma glucose and/or glycated hemoglobin (HbA1c) could be considered, noting significantly reduced accuracy” [12].

The rationale for using these tests is based on their role in diabetes diagnosis, their ease of administration, and the extensive body of research linking elevated glucose to long-term complications such as retinopathy, renal disease, neuropathy, and vascular disease [20,46,47]. All of the testing methods were introduced into clinical practice due to the belief that they would provide improvements in patient care, despite very limited supportive evidence. The current adherence to glucose-centric testing using the OGTT, fasting BSL, and HbA1c testing has impeded progress in the early diagnosis and prevention of metabolic dysfunction and complications of IR, hyperinsulinaemia, and hyperglycaemia in women with PCOS [19,20].

### 3.3. Treatments Based on the Glucose-Centric Model

Treatment approaches within the glucose-centric model of PCOS are largely derived from strategies used in T2DM prevention and management. They typically aim to delay the onset of overt hyperglycemia in IGT or reduce elevated blood glucose levels in T2DM. First-line recommendations include lifestyle advice focused on weight loss, increased physical activity, and diet [12]. Diet composition is suggested to be consistent with population guidelines for sustainable healthy eating, tailored to individual preferences, and goals.

Pharmacological treatment for impaired blood glucose levels and T2DM in women with PCOS includes metformin, an insulin-sensitizing agent with glucose-lowering effects [48]. Metformin has multiple mechanisms of action that improve IR, symptoms of PCOS, and fertility [49]. Inositol supplementation is now considered a treatment option as it may improve metabolic and fertility outcomes [12].

### 3.4. Strengths of the Current Approach

The glucose-centric approach can be effective in reducing long-term complications in women with PCOS who have already developed IGT or T2DM [24]. Evidence supports the role of weight loss, dietary modification, and metformin in improving metabolic outcomes and reducing the risk of T2DM in this subset of patients [24,49,50].

Moreover, the glucose-centric model aligns with existing public health frameworks, particularly those aimed at identifying and managing prediabetes and T2DM [51]. For example, glucose-based screening integrates easily into primary care settings [45], facilitating early detection of overt glycemic abnormalities in high-risk PCOS populations [52]. Clinicians are generally familiar with interpreting these tests, and treatment pathways are clearly defined in national and international guidelines.

### 3.5. Limitations and Clinical Consequences of the Translation Gap

By focusing primarily on glycemic thresholds, the glucose-centric model overlooks the early and often silent stage of IR and hyperinsulinemia that drives many of the reproductive, metabolic, and inflammatory manifestations of PCOS [48]. The disconnect between pathophysiological understanding and clinical assessment reflects a broader translation gap. While the research literature consistently identifies IR as a central mechanism in PCOS [2], clinical guidelines and routine practice continue to emphasize glucose assessment and management [12].

In addition, the outdated assumption that T2DM in PCOS is an inevitable and inherited progressive disease, rather than a largely preventable consequence of untreated IR, has delayed recognition of the underlying metabolic problem and hindered opportunities for early targeted intervention to prevent future chronic diseases. This belief contributes to an emphasis on managing blood glucose rather than addressing the upstream metabolic drivers of IR and hyperinsulinemia. However, nowadays, there is growing evidence that lifestyle modification and early targeted interventions can significantly reduce the risk of disease progression [50]. An insulin-centric strategy allows for earlier detection and more proactive management, potentially preventing both GDM and T2DM [53].

## 4. Overview of Insulin’s Diverse Biological Actions

Insulin plays a central role in the regulation of human metabolism, energy storage, appetite regulation, immune function and inflammation, hormone regulation and reproduction, and vascular dynamics and blood pressure regulation (Table 1) [54,55,56,57,58,59,60,61,62,63]. Insulin facilitates glucose removal from the blood in insulin-dependent tissues such as skeletal muscle, cardiac muscle, adipose tissue, and endothelium, and also acts as an anti-inflammatory hormone.

### 4.1. Cellular Actions of Insulin

Insulin is a peptide hormone only produced and secreted by the beta cells in the Islets of Langerhans of the pancreas [55,68]. Insulin binds with the extracellular domain of the alpha subunit of the insulin receptor, which induces autophosphorylation of tyrosine kinase on the intracellular side of the membrane. This initiates a cascade of signal transduction events via two key pathways that lead to different distal signaling responses in target tissues. These include the phosphatidylinositol-3 kinase (PI-3K) metabolic pathway, which activates transcription factors such as forkhead box 01, tuberous sclerosis complex 1/2, sterol regulatory binding protein 1c, and the mitogen-activated protein kinase (MAPK) pathway that activates cell growth and proliferation [55,69].

Activation of the PI-3K pathway induces GLUT4 translocation to the cellular membrane in insulin-dependent tissues such as muscle and adipose tissue [70]. The PI-3K signaling cascade can upregulate the transcription of key steroidogenic enzymes, including CYP17A1 (17α-hydroxylase/17,20-lyase), which play a central role in converting androgenic precursors (pregnenolone and progesterone) into androgens (testosterone and androstenedione) [71].

### 4.2. Tissue-Specific Actions of Insulin

Insulin is an anabolic hormone that regulates a myriad of tissue-specific cellular processes, such as protein, fat, glucose, and glycogen synthesis, RNA and DNA synthesis, as well as cellular proliferation and differentiation. Importantly, insulin inhibits catabolic processes by inhibiting glucagon release from the pancreas, gluconeogenesis in the liver, proteolysis in muscle, and lipolysis in adipose tissue [55]. In this way, insulin acts as a metabolic switch between anabolic and catabolic pathways to control energy production, storage, and utilization during feeding and fasting [72].

### 4.3. Anti-Inflammatory Actions of Insulin

Beyond metabolism, insulin links energy regulation and immune modulation, contributing to adaptive survival responses to environmental stressors [73]. Insulin inhibits transcription factor NF-kB and reduces the production of inflammatory cytokines [58], inhibits NRL inflammasome formation [59], decreases leukocyte adhesion to the endothelium [60], and prevents hyperglycaemia-induced production of reactive oxygen species and advanced glycation end products [57]. Under conditions of insulin sensitivity, the anti-inflammatory effects of insulin may have an evolutionary protective function to prevent overactivation of the immune system to small amounts of ingested antigens that cross the gastrointestinal barrier during food intake [74]. Therefore, insulin may be part of an extensive network of communication mechanisms that contribute to the systemic regulation of the inflammatory response [2,75,76]. In contrast, IR is pro-inflammatory and part of an adaptive survival response to a variety of environmental challenges (see Section 5) [77,78,79].

## 5. The Adaptive Significance of Reduced Insulin Sensitivity and Insulin Resistance in PCOS

### 5.1. Insulin Sensitivity as a Continuous Variable

Insulin sensitivity reflects the ability of insulin to remove glucose from the blood and restore normoglycaemia [31]. Decreased insulin sensitivity is a continuous variable that is classified as IR once it reaches an arbitrarily defined cut-off value, during an experimental hyperinsulinemic-euglycemic clamp test (e.g., 4.45 mg/kg/min) [80], or as an elevated surrogate marker test in clinical practice [81]. The functional consequences of reduced insulin sensitivity manifest as altered tissue responsiveness to insulin. This is particularly relevant in PCOS, where a systematic review of clamp studies reported that women with PCOS exhibit a 27% reduced sensitivity to insulin [7].

Despite the widespread use of the term IR, as yet, there is no universally agreed upon normal range for use in clinical practice. Variability in diagnostic cut-off values contributes to inconsistencies in reported prevalence rates of IR in PCOS [82]. Functionally, IR refers to a diminished biological response to insulin stimulation in target tissues that may reflect a physiological adaptation or a pathological condition, depending on the context [8].

### 5.2. Physiological Insulin Resistance as an Adaptive Survival Mechanism

Decreased tissue sensitivity to the physiological actions of insulin has become synonymous with the pathological effects of IR, despite the fact that reduced sensitivity to insulin is an evolutionarily conserved homeostatic survival mechanism [1,4,8,83]. Reduced insulin sensitivity appears to be a key inherited component in PCOS that improves survival in response to a range of internal and external environmental situations [1,84]. Physiological IR and hyperinsulinemia occur during systemic infection, trauma, starvation, adolescence, and pregnancy, and limit glucose uptake in insulin-dependent tissues, such as muscle and adipose tissue [1,8,17,78]. IR therefore functions as an adaptive mechanism to redistribute glucose to tissues in need, such as immune cells, brain, bone, and the fetus [17,77].

From an evolutionary perspective, women with PCOS can be considered “metabolically elite”, as they can store energy efficiently and redirect glucose to tissues with increased demand for energy.

### 5.3. The Shift to Pathological Insulin Resistance

Insulin resistance becomes pathological when the adaptive reduction in insulin sensitivity persists or is exaggerated in response to modern environmental stressors. In this state, metabolic dysfunction arises from disrupted insulin signaling pathways, often triggered by recurrent diet-induced hyperglycemia and hyperinsulinemia, metabolic intermediates, chronic stress, inflammatory cytokines, hormonal imbalances, and exposure to endocrine-disrupting chemicals [55,85].

Pathological IR is always associated with hyperinsulinemia, which acts together and contributes to the symptoms, biochemical, metabolic, immune, and reproductive features of PCOS (see Section 6) [1]. Hyperinsulinemia is an early indicator of metabolic dysfunction and is associated with many genetic, nutritional, and environmental factors [86]. Dysregulation of insulin biology is also a key pathophysiological component of the initiation and progression of complications and chronic disease in women with PCOS [13,15,87].

## 6. Insulin Resistance and Hyperinsulinemia as Central Drivers of PCOS and Related Complications

### 6.1. Bidirectional Relationship Between Insulin Resistance and Chronic Inflammation

The pathogenesis of PCOS in contemporary populations is thought to be due to the epigenetic effects of nutritional, environmental, and lifestyle exposures on inherited adaptive gene variants [1,2,4,5,6,7,8,9,10,11]. Chronic low-grade systemic inflammation and IR are central drivers of the pathophysiology of PCOS [2,88,89]. The bidirectional relationship between IR and chronic inflammation creates a vicious cycle that exacerbates both the metabolic and reproductive disturbances seen in women with PCOS.

Inflammation is an evolutionary-conserved adaptive survival response of the immune system primarily directed at combating infection, toxins, allergens, and tissue injury [90]. Inflammation can be physiological or pathological, and optimal health is achieved by balancing anti- and pro-inflammatory effects aimed at removing infected, damaged, or aging cells [91]. Inflammatory cytokines, chemokines, and extracellular vesicles help support a successful protective response by connecting neuroendocrine and immunometabolic systems [92,93,94].

As part of this response, inflammation induces a rapid adaptive metabolic response by downregulating insulin signaling and GLUT4 translocation, resulting in reduced insulin sensitivity and glucose uptake, IR, and increased serum glucose levels [95]. This is a physiological response to redirect energy to immune cells and vital organs [96]. As a result, immune cells can increase their energy demand from 10% of basal energy use to 30% when required [97]. In addition, inflammation and metabolic adaptation are linked to reproduction to optimize fertility [98]. Consequently, both IR and inflammation can increase ovarian androgen production, which augments IR and downregulates ovulation [99,100].

Women with PCOS are believed to have an evolutionary beneficial “pro-inflammatory design” that results in a heightened physiological response [8,101]. In a modern environment, poor-quality diet and a range of environmental and lifestyle factors result in chronic activation of the immune system and metaflammation [102]. The combined effects of IR and chronic low-grade inflammation contribute to the symptoms, biochemical and endocrine features, and progressive metabolic diseases associated with PCOS [1,2].

IR can cause inflammation via direct or indirect mechanisms. IR is accompanied by reduced uptake and oxidation of glucose, which results in hyperglycaemia, which in turn triggers oxidative stress [103]. Oxidative stress activates innate intracellular defense systems, such as the endoplasmic reticular stress response and inflammasome formation, that initiate pro-inflammatory cytokine production and inflammation [94]. Hyperglycaemia-associated advanced glycation end-products (AGEs) cause oxidative stress and inflammatory cytokine production, which are protective if short-lived and limited, and pathological if chronic and excessive [104,105]. The bidirectional relationship between IR and chronic inflammation ensures a coordinated and cooperative physiological response to stressors, which become self-reinforcing and pathological following excessive activation from nutritional, environmental, and lifestyle factors in the contemporary environment.

### 6.2. Insulin Resistance Disrupts Ovarian Function

Ovarian tissue remains sensitive to insulin even in the presence of hyperinsulinemia associated with IR [89]. This heightened ovarian sensitivity may reflect an adaptive mechanism to suppress ovulation under conditions of metabolic stress or scarcity [2]. A maladaptive consequence is that the chronically elevated insulin levels associated with pathological IR can significantly disrupt ovarian function.

Insulin directly stimulates androgen production by theca cells via the PI-3K and MAPK pathways and through the inositolglycan signal transduction system [64,71,106]. Insulin also increases the amplitude of gonadotropin-releasing hormone (GnRH)-stimulated LH pulses [107,108] and acts synergistically with LH to enhance androgen synthesis, primarily via direct activation of ovarian insulin and IGF-1 receptors [64]. Insulin also promotes serine phosphorylation of insulin receptor substrates and possibly LH receptors, amplifying the androgenic response of theca cells even in the absence of high LH levels [109]. In parallel, insulin suppresses hepatic production of sex hormone binding globulin, increasing the bioavailability of circulating androgens and further enhancing their activity in target tissues [110].

Hyperinsulinemia and IR also disrupt ovarian function indirectly through oxidative stress and inflammatory pathways. Hyperglycemia-associated AGEs are elevated in women with PCOS and IR [111] and accumulate in the follicular environment, impairing steroidogenesis and disrupting granulosa cell function [2,112,113]. Together with hyperinsulinemia, AGEs contribute to mitochondrial oxidative stress, diminished oocyte quality and maturation, and ovulatory disturbance [89,114].

Additionally, IR alters ovarian adipokine signaling by reducing adiponectin and increasing leptin. These changes impair granulosa and theca cell function and promote a pro-inflammatory, insulin-resistant local environment [50]. Insulin may increase anti-Müllerian hormone (AMH) production by granulosa cells, potentially contributing to follicular arrest, the accumulation of small antral follicles, and ovulatory disturbance [115]. Another important mechanism involves insulin-mediated impairment of follicle-stimulating hormone (FSH) signaling in granulosa cells, leading to downregulation of aromatase expression [33]. Reduced ovarian aromatase activity decreases ovarian estradiol production and contributes to androgen excess and ovulatory dysfunction, which are all hallmarks of PCOS [116].

### 6.3. Insulin Resistance Induces Neuroendocrine Disturbance and Dysregulates the Hypothalamic-Pituitary-Ovarian Axis

The hypothalamic-pituitary-gonadotropin system plays a central role in the regulation of reproduction by integrating different neuroendocrine, metabolic, and environmental signals [117]. GnRH secretion is regulated by feedback loops involving gonadal hormones, primarily estrogen and progesterone, which exert both stimulatory and inhibitory effects on the hypothalamic-pituitary-ovarian (HPO) axis and maintain cyclical reproductive function [118]. Insulin receptors are expressed in the hypothalamus, particularly on kisspeptin and pro-opiomelanocortin neurons, where they contribute to the regulation of GnRH pulsatility and energy homeostasis [108,119]. Under conditions of normal insulin sensitivity, these pathways support coordinated reproductive and metabolic function. In states of IR, however, central insulin signaling is disrupted, leading to altered GnRH neuronal activity, increased amplitude of GnRH-stimulated LH pulses, and impaired or absent ovulation [89,120]. The absence of ovulation further dysregulates GnRH/LH pulsatility by removing the negative feedback normally provided by luteal-phase progesterone [121].

In a normal ovulatory cycle, progesterone and its neurosteroid metabolites, such as allopregnanolone, interact with GABA-A receptors on kisspeptin neurons to inhibit GnRH secretion and stabilize the HPO axis [121,122]. This inhibitory action is diminished by androgens, which can bind to hypothalamic progesterone receptors [123], but enhanced by ovulatory levels of estradiol, which upregulate progesterone receptor expression in kisspeptin neurons and stimulate astrocytes to synthesize neuroprogesterone [124,125]. Hypothalamus-derived estradiol (neuroestradiol) also contributes to GnRH regulation [126]. In the absence of these regulatory brakes (due to anovulation, decreased estrogen, and impaired hypothalamic progesterone inhibition), a feedforward loop develops where elevated LH promotes further androgen excess, which exacerbates IR and perpetuates anovulation [127]. By impairing ovulation and interrupting the normal maturation to ovulatory cycling, IR may therefore exaggerate or prolong hyperandrogenism and anovulatory cycles. This may also be a factor that drives the onset of neuroendocrine dysfunction in adolescence and contributes to the onset of symptoms of PCOS [128,129].

In summary, the pathophysiology of PCOS involves a vicious cycle between hypothalamic-pituitary dysfunction and disrupted ovarian steroidogenesis. Increased GnRH pulse frequency leads to elevated LH and relatively low FSH, which results in excess ovarian androgen production. Decreased ovarian aromatase function, and possibly accelerated degradation of estradiol in ovarian follicles, lead to lower estradiol levels and hyperandrogenism [130,131]. The lower estradiol and elevated androgens, coupled with elevated AMH, contribute to follicular arrest, anovulation, and polycystic ovarian morphology [115]. Anovulation perpetuates the neuroendocrine disruption by preventing the luteal-phase rise in estradiol and progesterone, and the negative feedback needed to regulate GnRH output.

As discussed above and in Section 6.2, IR and hyperinsulinemia disrupt neuroendocrine regulation in many ways and are the primary drivers of HPO dysfunction. As a result, lifestyle-based interventions are the first line of treatment recommended in the 2023 International Guidelines and in phase 1 of the insulin-centric model (see Section 7.4) [12]. Second-line pharmacotherapeutic support may also be required to counteract the effects of IR and hormonal imbalances if lifestyle measures alone are insufficient (discussed in Section 7.5). While the combined oral contraceptive pill (COCP) has long been used to manage PCOS symptoms, it suppresses ovulation and disrupts natural cyclical hormone production. In addition, the OCP, particularly those containing androgenic progestins, has a risk of side effects such as thromboembolism [132] and may worsen IR [133]. Emerging alternatives include cyclical human-identical estradiol (17B-estradiol patch or gel) and oral luteal-phase micronized progesterone [134,135,136]. Although some small studies report promising results, conflicting results have been reported [137], and large-scale randomized trials are required.

### 6.4. Bidirectional Relationship Between Insulin Resistance and Hyperandrogenism

Androgens have a physiological role in developmental programming [6], adipose tissue differentiation [138], body fat distribution [139], skeletal muscle growth [140], hypothalamic control of food intake and energy balance [141], energy metabolism [142], and insulin signaling in insulin-targeted tissues [143]. Increased androgen levels in women with PCOS can result in pathological effects in any of these tissues or physiological systems. Excess androgens promote beta-cell dysfunction and impaired insulin secretion, disruption of insulin signaling in muscle and adipose tissue, and an increase in visceral adipose tissue [144].

There is clear evidence that elevated androgens can exacerbate IR, and both hyperinsulinaemia and IR can lead to elevated androgens [109,114]. Nevertheless, mildly elevated androgen levels may have a range of adaptive survival functions, such as increased physical strength and decreased reproductive function in times of environmental stress [1,4,84]. In addition, the augmentation of IR by elevation of androgens would also provide other survival advantages, such as the redirection of energy to tissues of need [145]. The bidirectional relationship between insulin signaling pathways and androgens may represent a self-reinforcing feedback loop that links metabolic health to optimal reproductive performance.

Although reducing androgen levels would be expected to improve insulin sensitivity, conflicting results have been reported. Several small clinical trials, including studies using GnRH analogs and androgen receptor antagonists such as flutamide, have reported inconsistent effects on insulin sensitivity, with many showing no improvement despite reduced androgen levels [146,147,148,149,150,151,152,153]. Surgical interventions such as ovarian drilling and oophorectomy similarly yielded mixed results [154,155]. These findings are sometimes interpreted to mean that androgens are not the primary cause of IR. However, the interpretation of these conflicting reports is limited by the inclusion of small numbers of patients, the use of indirect methods of assessing insulin sensitivity, and the short duration of treatment in some studies. In addition, the interpretation of the GnRH analog studies should consider potential compensatory metabolic changes resulting from the estradiol deficiency and neuroendocrine disruption induced by these medications.

Further evidence that hyperinsulinemia and IR are primary causes of hyperandrogenism comes from studies investigating inherited severe insulin resistance syndromes (Rabson-Mendenhall syndrome, type A, B, and C insulin resistance syndromes, and lipodystrophies) [156]. Ovarian hyperandrogenism is present in many of these individuals and is secondary to IR. A prospective study of 31 women with PCOS randomized to 3 treatment groups (flutamide, metformin, or flutamide/metformin) for 9 months, found that combined treatment with flutamide/metformin resulted in greater improvements in lipid profiles, androgen levels, and IR, than with monotherapy alone [157]. Finally, a meta-analysis of 24 randomized trials reported a beneficial impact of using the insulin-sensitizing medication metformin to reduce both metabolic parameters and serum androgen levels [158].

In summary, although elevated androgen levels clearly exacerbate IR, intervention trials where androgen levels are reduced do not consistently improve IR. This is likely due to the fact that IR has a number of underlying causes, such as poor quality diet, gastrointestinal dysbiosis, chronic low-grade inflammation, stress, and circadian disruption, in addition to elevated androgens [2]. On the other hand, improving insulin sensitivity can significantly improve androgen levels and PCOS-related symptoms [158]. Evidence suggests that both IR and chronic inflammation are primary drivers of hyperandrogenism via specific molecular effects in the hypothalamus, ovaries, liver, and metabolic pathways [99,100]. IR and hyperandrogenism reinforce each other, perpetuating both metabolic and reproductive dysfunction. Understanding this bidirectional relationship is key to devising a combined therapeutic strategy to reduce both IR and elevated androgens, in order to provide the most beneficial treatment approach.

### 6.5. Adverse Effects of Insulin Resistance on the Endometrium, Placenta, and Associated Pregnancy Complications

The key pathophysiological features of PCOS (chronic inflammation, IR, and hyperandrogenism) have all been individually associated with adverse endometrial and decidual changes that contribute to altered placental development and function [13,159,160,161,162,163,164,165]. The resulting dysfunctional cellular network at the maternal–fetal interface has an adverse impact on bidirectional communication between maternal decidual cells and fetal trophoblast cells involved in the formation of the placenta [17,166,167,168]. As a consequence, women with PCOS are at significantly increased risk of miscarriage and implantation failure and have reduced success rates following assisted fertility treatments [169,170]. In addition, women with PCOS have an increased risk of all of the “great obstetrical syndromes” (spontaneous preterm labor, fetal growth restriction, stillbirth, and preeclampsia), which share common pathophysiological processes and placental abnormalities [171,172,173]. In addition, women with PCOS have a significantly increased incidence of GDM, related to their underlying IR [174].

A range of possible mechanisms has been proposed to explain the effect of preexisting hyperinsulinaemia and IR on placental development as a result of in vitro, animal, and human studies. Insulin can inhibit aromatase activity in human trophoblasts [175], which may connect hyperinsulinaemia to excess placental androgens. The majority of reported studies show an adverse impact of maternal hyperandrogenism on pregnancy complications [17,165,176]. Elevated insulin was shown to cause DNA damage, apoptosis, and reduced cell survival in trophoblasts in vitro [161], which could contribute to impaired trophoblast migration and spiral artery remodeling. Pretreatment of trophoblasts with metformin prevented insulin’s deleterious effects in a mouse model [177]. IR has been associated with altered transcriptome signatures in pathways that may affect placental development [162]. Interestingly, altered endometrial transcriptome signatures isolated from a variety of endometrial cells were reversed following 16 weeks of treatment with metformin and lifestyle, in obese women with PCOS, hyperandrogenism, and IR [178]. Placental trophoblasts from obese women are significantly less sensitive to insulin than non-obese women, and obese women have greater placental lipid accumulation (fatty placenta), similar to IR-related maternal lipotoxicity [179,180]. This may contribute to placental inflammation and metabolic and nutrient transport abnormalities.

Adherence to a healthy lifestyle (higher diet quality, regular exercise, maintaining normal weight, non-smoking, and avoidance of alcohol) has been found to reduce the risk of pregnancy complications in women with PCOS [181,182], and is cost-effective [183,184]. Accumulating clinical and molecular evidence therefore suggests that IR and hyperinsulinaemia contribute to abnormal placental development and pregnancy complications that can be prevented or minimized with preconception and antenatal lifestyle and medical interventions.

## 7. Introduction of an Evidence-Based Insulin-Centric Model of Insulin Resistance in PCOS

### 7.1. Rationale for Changing from a Glucose-Centric View of Glycaemic Disturbance to an Insulin-Centric Model

The limitations of the glucose-centric approach for early diagnosis and prevention of complications of T2DM have been recognized for decades [185,186,187]. Historically, current testing and management protocols facilitated the detection of hyperglycemia and interventions designed to reduce premature morbidity and mortality. However, these interventions tend to occur late in the disease process after 30–50% of individuals diagnosed with T2DM have already developed complications [20]. As a result, there is now a growing consensus that a new approach is necessary for early detection of metabolic precursors to overt T2DM and related complications, such as beta cell failure, IGT, and IR [188]. These approaches encompass novel diabetes classification systems [189], diagnosis of diabetes using machine learning (ML)-based predictive models [190], optimized diabetes detection ML models utilizing feature engineering and ensemble learning [191], and interactive network models [192]. They also include a dysglycemia-based framework for managing multi-morbidity [193] a comorbidity-centric algorithm [194], and strategies that target the “ominous octet” to prevent damage across multiple organs [195,196]. In addition, there are diverse models centered on different aspects of the disease, which include glucagonocentric [197], cardiorenal-metabolic [198], TOR-centric [199], beta cell-centric [200], adipocentric [201], or insulin-centric approaches [202,203,204,205].

A Delphi survey of 26 consensus statements prepared by the Expert Group on Inositol in Basic and Clinical Research and on PCOS (EGOI-PCOS) recommended that metabolic factors such as IR should be included in clinical guidelines on PCOS [203]. Similarly, a 2024 review by a large group of international authors concluded that the diagnostic criteria for PCOS would be greatly improved by the inclusion of metabolic alterations such as IR [204]. These recommendations are based on evidence that correlates the level of IR with the severity of PCOS [206]. In addition, it is recognized that IR is a precursor for metabolic disorders such as GDM, metabolic syndrome, and T2DM, particularly in hyperandrogenic phenotypes of PCOS [204,207,208].

**Figure 3:**
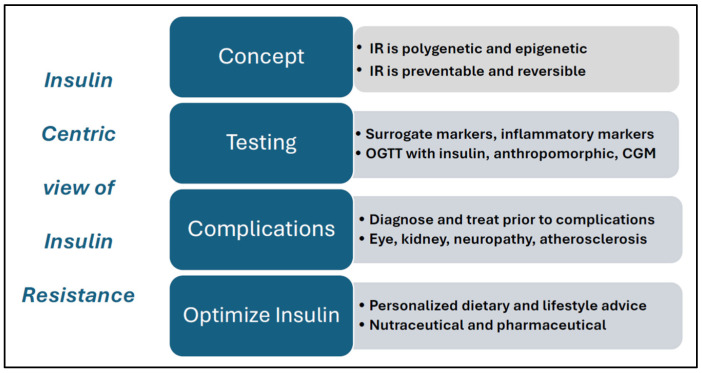
Features of an insulin-centric model of insulin resistance. Abbreviations: IR = insulin resistance; OGTT = oral glucose tolerance test; CGM = continuous glucose monitoring.

Many women show no symptoms of hyperglycemia and are diagnosed with T2DM after presenting with other medical problems or during a routine check-up [209]. Adolescents and women with PCOS represent an ideal group for early detection and intervention. They usually present at a young age with apparently non-metabolic symptoms, such as menstrual disturbance, acne, hirsutism, or infertility, and have a high likelihood of underlying metabolic disturbance and IR [2,7]. As a result, we advocate for incorporating an insulin-centric approach in the assessment and management of PCOS (Figure 3). This method builds on the well-established role of hyperinsulinemia and IR in metabolic dysfunction and hyperandrogenism, thereby enabling earlier interventions to prevent progressive morbidity and premature mortality [210]. One advantage of introducing an insulin-centric approach is that available methods for assessing hyperinsulinemia and surrogate markers of IR could easily be incorporated into current management protocols.

### 7.2. Reasons for Delayed Introduction of an Insulin-Centric Paradigm

Despite widespread acknowledgment of the shortcomings of the glucose-centric paradigm and the possible clinical benefits of an insulin-centric approach, significant barriers hinder changing current practices. These challenges include the inherent physiological complexity and incomplete understanding of insulin biology and IR, difficulties with measurement, standardization, and test validation, gaps in the evidence that contribute to outcome uncertainty, and a persistent clinical inertia that resists altering long-established paradigms [211].

We assert that the current evidence robustly supports shifting to an insulin-centric model. Although our understanding of insulin dynamics and supporting data may have limitations, the overwhelming clinical need for change outweighs these gaps. It is important to note that current glycemic tests were not developed through exhaustive, long-term intervention trials, yet their timely adoption has led to significant improvements in patient care. Nevertheless, it is now widely appreciated that the glucose-centric approach has significant limitations [185,186,187,189,190,191,192,193,194,195,196,197,198,199,200,201]. Clinicians accustomed to relying on “clinical judgment” need to embrace “evidence judgment” when there is a significant unmet need, despite an imperfect evidence base.

### 7.3. Testing for Insulin Resistance—Measurement Challenges and Standardization

#### 7.3.1. Hyperglycaemic–Euglycaemic Clamp Test

The hyperglycaemic–euglycaemic clamp (clamp) test is the gold standard procedure for measuring IR [212]. The clamp test involves simultaneous infusions of high-dose intravenous insulin and glucose in order to suppress hepatic gluconeogenesis and create a steady state blood glucose level (5.5 mmol/L) [213]. Insulin sensitivity is measured over a normal biological range, and IR is diagnosed when the whole-body glucose disposal rate reaches an arbitrary cut-off of 4.9 mg/min/kg [80] (or 46.0 ± 16.9 micromol/min/kg lean body mass) [214].

The clamp test is only suitable for experimental studies in research centers, due to the complexity, associated risks, expense, and time required to conduct the procedure [213]. The clamp test has considerable intra-laboratory variability due to different methods of measuring glucose and insulin, and is operator dependent. In addition, the clamp test underestimates glucose disposal in very IR participants due to incomplete suppression of hepatic gluconeogenesis [213]. When taken together, it is clear that the clamp test has many limitations, even if it were suitable for use in clinical settings. Despite these limitations, the clamp test is used as a gold standard to compare the accuracy of surrogate marker tests.

#### 7.3.2. Surrogate Biomedical Markers of Insulin Resistance and Hyperinsulinemia

Fasting insulin levels are useful to provide preliminary information about insulin dynamics, but may differ widely within individuals, can have variable performance characteristics at low concentrations (<12 pmol/L), may be impaired by the presence of insulin antibodies, and assay standardization has not been determined internationally [215,216]. Nevertheless, consistently high levels of fasting insulin reflect underlying IR.

Insulin assays are more accurate predictors of IR when combined with other markers [216]. A wide range of surrogate marker indices have been compared to the clamp test to determine accuracy, precision, cutoff values, and reproducibility. It is not the purpose of this review to describe these in detail, and we refer the reader to current comprehensive reviews [23,216,217]. Examples include Homeostatic Model Assessment-IR (HOMA-IR), HOMA-Triglyceride index (HOMA-TG), Quantitative Insulin Sensitivity Check index (QUICKI), TG/High Density Lipoprotein (TG/HDL) index, TG/Glucose (TG/G) index, alanine aminotransferase/aspartate aminotransferase (ALT/AST) ratio, and Adipose-IR index. Many of these indices have been shown to be valuable predictors of IR. The most commonly used and tested index is the HOMA-IR. A recent Delphi consensus statement found that 72% of gynecologists and endocrinologists agreed that a HOMA-IR ≥ 2.5 is sufficient to define IR [203]. In addition, HOMA-IR could be coupled with circulating inflammatory mediators known to be associated with IR, adipose-tissue derived biomarkers, or other emerging novel molecules, to improve diagnostic accuracy and predictive ability [23,218].

Insulin resistance is a global health problem that is associated with significant clinical symptoms and is an early predictive indicator of future chronic disease in women with PCOS [23]. There is an urgent worldwide need for the development of accurate measurement, risk prediction, and evaluation of how surrogate indices change in intervention trials. In order to facilitate a rapid transition from a glucose-centric approach to an insulin-centric paradigm, an international collaborative effort is required to determine a consensus approach to surrogate marker testing, based on the best available evidence. The current evidence base far exceeds that used during the historical introduction of glucose-based testing (discussed in Section 3.1 and Section 3.2). Clinicians should be informed about the limitations of using surrogate marker indices and learn to interpret the results in the context of other tests, such as dynamic glucose-insulin testing, continuous glucose monitoring, anthropomorphic data, inflammatory cytokines, and other biomarkers. These assessment tools should be used in collaboration with individual clinical history and examination, as is usually performed during a comprehensive personalized medical assessment. Future research should focus on investigating the correlation between surrogate indices, biomarkers, and clinical outcomes.

#### 7.3.3. Dynamic Glucose-Insulin Testing

Dynamic glucose-insulin testing is a modified OGTT where both glucose and insulin are measured at specified intervals (e.g., 0, 30, 60, 90, and 120 min) [219]. Measurement of insulin at the same time as glucose facilitates a dynamic assessment of the magnitude of insulin response following a standardized glucose load (e.g., 75–100 g) [220]. This timing allows for assessment of the early hyperinsulinemic response pattern, highlighting abnormalities in insulin secretion that static tests might miss [221,222].

In women with early IR, the pancreas may overcompensate by secreting increased insulin at the 30 min point of the OGTT. This hyperinsulinemic response helps compensate for reduced insulin sensitivity in muscle, fat, and liver tissues, keeping blood glucose levels normal even when the metabolic system is under stress. Therefore, dynamic glucose-insulin testing allows for a concurrent assessment of insulin secretion with insulin sensitivity and can provide a window into the early pathological changes that precede overt dysglycemia [220,223].

#### 7.3.4. Anthropomorphic Data

Anthropomorphic data aim to assess body composition and fat distribution as a surrogate indicator of metabolic and endocrine disturbance and future risk prediction in PCOS [224]. Commonly used metrics include BMI, waist circumference, waist–hip ratio, waist–height ratio, visceral adiposity index (VAI), and more detailed fat mass assessments with Dual X-ray Absorptiometry (DEXA) scans and Magnetic Resonance Imaging (MRI). When coupled with biomedical surrogate markers, anthropomorphic data can help construct a comprehensive profile of metabolic status and risk. Waist-to-height ratio is emerging as a better predictor of hyperandrogenism than BMI, in women with PCOS [225]. VAI is a gender-specific index, based on anthropomorphic and metabolic parameters, and has been found to correlate with visceral adipose dysfunction and IR in women with PCOS [226].

Multidimensional analysis using anthropomorphic data, surrogate biomedical markers, and comprehensive metabolic assessments could further refine risk stratification and treatment monitoring, especially in response to lifestyle interventions. Prospective interventional studies are required to assess the value of combined indices in clinical management and future chronic disease risk prediction.

#### 7.3.5. Concurrent Testing for Inflammatory Markers

Large systematic reviews confirm the important role of chronic low-grade systemic inflammation as a key pathophysiological process that acts together with IR in the pathogenesis of PCOS [227,228,229]. The dysbiosis of gut microbiota theory of the pathogenesis of PCOS proposed that poor-quality diet results in increased release of lipopolysaccharide from Gram-negative bacteria, which traverses the gastrointestinal barrier and activates toll-like receptors on submucosal macrophages. This, in turn, activates the NF-kB signaling pathway and increases inflammatory cytokine production and secretion [229,230]. It is now recognized that systemic inflammation can be initiated at any mucosal surface [231], in response to microparticulate air pollution [232], microplastics [233], micro-organisms, other environmental antigens, and endogenous factors such as stress [234].

The bidirectional relationship and co-existence of IR and chronic inflammation (discussed in Section 6.1) support the measurement of inflammatory mediators as an aid to the assessment and management of IR in women with PCOS. The most commonly measured markers are C-reactive protein (CRP) and white blood count (WBC) [235]. Other markers of inflammation, such as interleukins, tumor necrosis factor, and homocysteine, are consistently elevated in PCOS, but are not routinely measured in clinical practice [236]. Large databases of the inflammatory proteome now exist (Olink multiplex inflammation panels and Immunology multiplex assay HCYTA 60K-PX48), and have been investigated in women with PCOS [237,238]. Measurement of inflammatory markers could provide a useful adjunct to the assessment of IR and warrant further investigation in clinical trials.

### 7.4. Targeted, Phase-Based Therapeutic Interventions: Phase 1-Lifestyle

It is important to educate patients about how IR and hyperinsulinemia impact both reproductive and metabolic health. Individual use of tools and training to self-monitor and adjust daily practices according to the feedback from their personalized assessments will result in empowerment and better autonomy [239]. Personalized dietary advice focused on low-glycemic foods that reduce post-prandial glucose levels will help facilitate better symptom control, weight management, and reduce future risk [240].

Personalized exercise prescriptions tailored to the abilities and interests of the individual will reduce visceral adiposity and increase compliance and quality of life [241]. behavioral and stress management strategies will reduce cortisol levels and improve hyperinsulinemia and IR [242]. Exercise and stress reduction, coupled with attention to sleep hygiene and circadian re-alignment, improve energy and motivation [243]. Increased emphasis on strengthening personal and community support systems may help reduce anxiety, depression, and loneliness [244].

### 7.5. Targeted, Phase-Based Therapeutic Interventions: Phase 2-Pharmacotherapeutic

A wide range of nutraceutical treatments have been studied for their ability to reduce IR and improve metabolic health [245]. These include resveratrol [246], N-acetyl cysteine [247], berberine [248], curcumin [249], magnesium [250], and inositol [12]. So far, only inositol has been recognized as a treatment option in the International Guidelines, but there is clearly a need for greater inclusion of many of these therapies in future clinical trials.

There is also a growing list of possible pharmaceutical treatment options for reducing IR that require more intensive investigation in women with PCOS. These include metformin, thiazolidinediones, alpha-glucosidase inhibitors, GLP-1 agonists, GIP and GLP-1 dual agonists, and SGLT-2 inhibitors [251]. It is likely that evidence from research in non-PCOS women will initially be translated to women with PCOS, but PCOS-specific trials are needed.

### 7.6. Targeted, Phase-Based Therapeutic Interventions: Phase 3-Monitoring and Support

Digital health tools, wearable technology, and continuous glucose monitoring can provide real-time insight and be integrated into patient management dashboards [252]. These technologies are readily available and widely used and need to be critically evaluated in clinical trials. Dynamic re-testing and data-driven lifestyle and pharmacotherapeutic adjustments should contribute to better patient education, compliance, and empowerment. Integrated metabolic reprofiling of glucose levels, lipid levels, inflammatory markers, and IR, coupled with diet and lifestyle reassessment, will contribute to the prevention of the health consequences of persistent IR [253].

### 7.7. Research and Continuous Improvement

The rapid introduction of wearable, digital, personalized monitoring technology is well ahead of rigorous clinical trial evaluation for safety, efficacy, and future disease prediction. These devices are promising tools for gathering large amounts of health data and can use ML to gain valuable insights that assist with individualized healthcare solutions [254]. Large-scale data collection using ML analytic capability should facilitate the timely refinement of the insulin-centric model. Interdisciplinary collaboration and longitudinal studies will be needed to ensure the model evolves in line with new scientific insights.

### 7.8. Active Surveillance of Future Pipeline Assessment Tools and Therapeutic Candidates

The establishment of cooperative interdisciplinary networks will be necessary to keep pace with the rapid nature of scientific progress in technically challenging medical and supportive disciplines. These include microbiome assessment [255], identification of microbiome-related metabolic signatures [256], genetic screening for high-risk single nucleotide polymorphisms (INSR, IRS-1/2, AR, and CAPN2) [257], epigenetic monitoring of methylation changes during treatment [258], and integrated multiomics assessments [259]. In addition, adaptive management platforms that continuously update and consolidate information from multiple sources will facilitate tailored adjustment to treatment strategies (mobile apps, online dashboards, or integrated clinical software) [260], and ML and AI-related tools [261]. There is an increasing need to assess the optimal balance between the use of digital technologies and human involvement in medical care [262].

## 8. Artificial Intelligence-Generated Insulin-Centric Model for the Assessment and Management of PCOS

**Figure 4:**
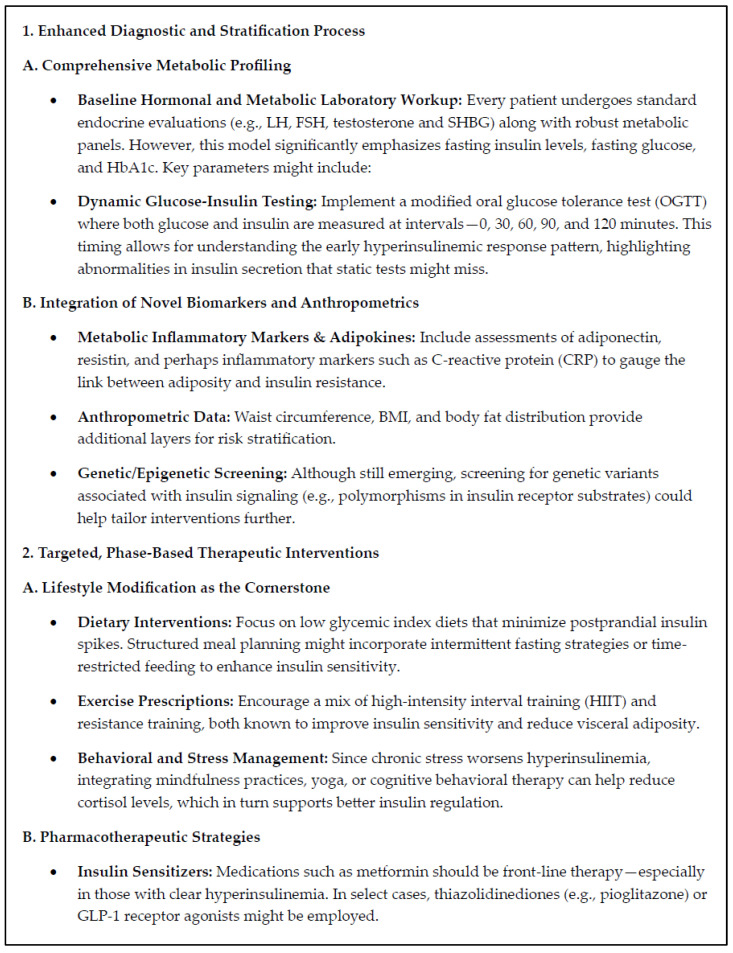

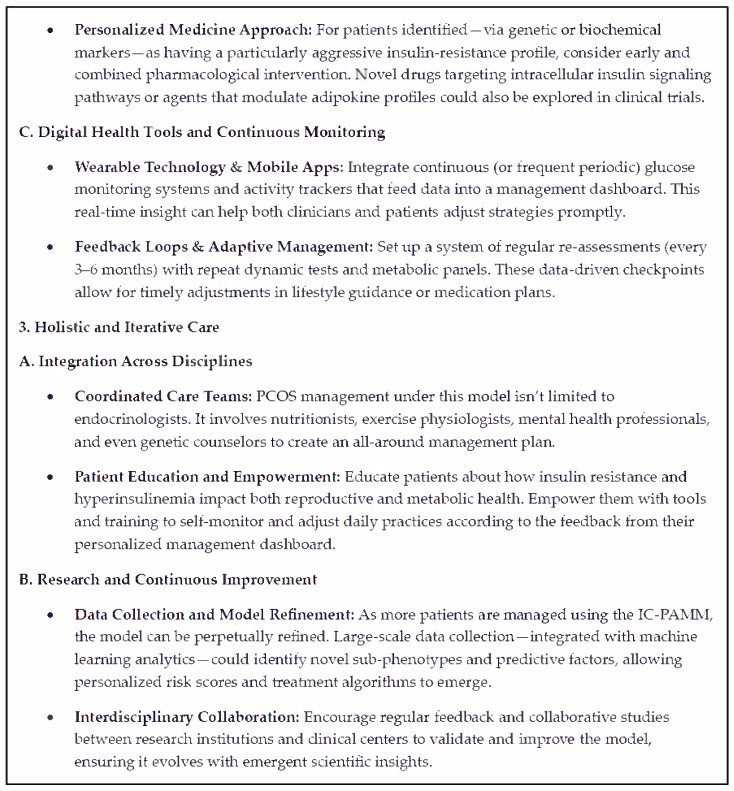
AI-generated future insulin-centric model for the assessment and management of polycystic ovary syndrome (PCOS): insulin-centric PCOS analysis and management model (IC-PAMM).

Below is a comprehensive proposal by the Generative Artificial Intelligence tool “Microsoft Co-pilot” for an insulin-centric model that rethinks the way we assess and manage PCOS. This approach builds on the well-established role of IR and hyperinsulinemia in driving androgen excess and metabolic and hormonal dysfunction in PCOS. The model—called the Insulin-Centric PCOS Analysis and Management Model (IC-PAMM)—frames insulin as both a diagnostic hallmark and a therapeutic target (Figure 4).

## 9. Discussion

We have combined a comprehensive review of the literature on historical aspects of the glucose-centric approach to the diagnosis of PCOS with an up-to-date discussion of insulin physiology, the adaptive significance of IR, and the role of IR and hyperinsulinemia in PCOS, with a state-of-the-art AI generated insulin-centric model for the assessment and management of PCOS (IC-PAMM). This novel model incorporates enhanced diagnostic testing, integration of new biomarkers and anthropometrics, targeted phase-based therapeutic interventions, and holistic and iterative care (Figure 4). We provide evidence supporting growing international interest in using currently available surrogate biomarkers, combined with other clinical assessment tools that focus on the identification of IR, to facilitate early intervention and prevention of progressive metabolic disease.

Insulin is a central regulatory hormone in human physiology, and there are believed to be insulin receptors on all human cells. Nevertheless, insulin exerts different actions in specific tissues and cells, which are reflected in the variety of physiological and pathological effects of IR and hyperinsulinemia. Hyperinsulinemia and IR are dynamically intertwined and always co-exist [253]. Hyperinsulinemia can cause IR, and IR can cause hyperinsulinemia. Reduced insulin sensitivity is a physiological adaptive survival mechanism that can become maladaptive in a modern environment [8]. An insulin-centric model recognizes altered insulin biology as not just a marker, but a primary driver of PCOS and its associated reproductive dysfunction and metabolic disease.

Most current therapies and guidelines target glycemic control, weight reduction, or broader metabolic improvements rather than focusing on reducing circulating insulin directly. An insulin-centric approach shifts the focus to reducing one of the primary upstream drivers of metabolic and endocrine dysfunction, rather than on downstream consequences. Changing a long-standing paradigm, even when it is recognized to have significant limitations, is always difficult and associated with significant barriers to change. While the evidence supporting an insulin-focused approach is complex and still evolving, it is nonetheless more substantial than the evidence that underpinned the adoption of the glucose-centric model. More importantly, there is a significant clinical need for a shift in focus from glucose to insulin to improve clinical management and quality of life and prevent premature morbidity and mortality.

The introduction of an insulin-centric model requires a coordinated international effort, as we have seen with the development of the International Guidelines for PCOS, to develop protocols based on existing evidence. Multidisciplinary collaborative research efforts will be required for data collection and model refinement to improve diagnostic strategies and develop shared therapeutic interventions that are integrated with digital health platforms and delivered in a holistic and personalized way.

## 10. Strengths and Limitations of the Current Review

### 10.1. Strengths

The current review provides a detailed discussion of the historical introduction of the longstanding glucose-based approach to the assessment and management of glycemic dysfunction in women with PCOS. The advantages and limitations of this model are explored and discussed in detail. The authors provide evidence and rationale that further supports the existing international momentum for a paradigm shift to a focus on insulin pathophysiology in PCOS. The report covers a broad range of complex topics that need to be examined when considering a change from a well-established traditional approach to an emerging new paradigm.

### 10.2. Limitations

There are many factors that limit the widespread acceptance of an insulin-centric approach to PCOS. PCOS is a heterogeneous syndrome that involves not only IR but also hormonal imbalances, such as hyperandrogenism and decreased estrogen and progesterone, inflammatory processes, disturbance of the microbiome, hypothalamic and ovarian alterations, and reproductive and psychological dysfunction. The pathophysiology of PCOS involves disturbance of a complex network of inter-related adaptive physiological systems, and there may be multiple initiating factors besides IR that drive symptoms and disease progression. This model is predicated on the hypothesis that IR and hyperinsulinemia are fundamental primary drivers of metabolic and reproductive dysfunction in women with PCOS.

## 11. Conclusions

PCOS is just the visible tip of a much larger global health crisis of metabolic-associated chronic disease. Early diagnosis in adolescents and young women allows for an in-depth assessment and management of the metabolic, hormonal, and psychological challenges that not only trigger symptoms but drive future health risks. By integrating comprehensive metabolic evaluations, dynamic insulin testing, and targeted lifestyle and medical interventions, this approach provides a versatile yet robust framework for tailoring PCOS treatment to the individual. IC-PAMM is a novel model that goes beyond simply correcting the biochemical imbalances, such as IR, chronic inflammation, and hyperandrogenism, to also enhance overall quality of life through holistic care. This multifaceted framework reflects an evolving understanding of PCOS that emphasizes early intervention and personalized treatment strategies, ensuring that care is both proactive and integrative.

## Figures and Tables

**Table 1:** Physiological actions of insulin.

Functions of Insulin	Mechanism	Reference
Pleiotropic cellular action	Tissue-specific action after binding to the insulin receptor	[54]
Energy storage	Adipose: glucose uptake, triglyceride storage, inhibits lipolysis	[55]
	Muscle: glucose uptake, glycogen synthesis, inhibits proteolysis	[55]
	Liver: glycogen synthesis, inhibits gluconeogenesis	[55]
Glucagon antagonist	Pancreas: paracrine suppression of glucagon release	[56]
Anti-inflammatory	BSL: helps keep BSL normal by decreasing ROS and AGE	[57]
	Inhibits NF-κB and MCP-1-activated cytokine production	[58]
	Reduced NLRP3 inflammasome formation and TLR signaling	[57,59]
	Reduced leukocyte adhesion to the endothelium	[60]
Kidney	Sodium reabsorption: water retention and volume expansion	[61]
	Reduced excretion of urate	[63]
Vasodilation	Arteriole: increased blood flow via endothelial nitric oxide	[62]
Tissue perfusion	Volume expansion and vasodilation	[61]
Blood pressure regulation	Volume expansion, vasodilation, and altered peripheral resistance	[61]
Ovary	Stimulates androgen synthesis via insulin and IGF-1 receptors	[64]
Central Nervous System	Hypothalamus: suppresses appetite, modulates energy expenditure, regulates GnRH pulsatility	[65]
	Liver: CNS-mediated regulation of hepatic glucose production	[66]
	Muscle: CNS-mediated promotion of glucose uptake	[67]
	Adipose: CNS-mediated suppression of lipolysis	[67]

Abbreviations: BSL = blood sugar level; ROS = reactive oxygen species; AGE = advanced glycation end products; NF-κB = nuclear-factor kappa B; MCP-I = monocyte chemoattractant protein-1; NLRP3 = nucleotide-binding leukocyte-rich, pyrin domain containing 3; TLR = toll-like receptor; IGF-1 = insulin-like growth factor-1; CNS = central nervous system.

## Data Availability

Not applicable.

## Abbreviations

AGE	Advanced Glycation End Products
ALT	Alanine Aminotransferase
AMH	Antimullerian Hormone
AR	Androgen Receptor
AST	Aspartate Aminotransferase
BMI	Body Mass Index
BSL	Blood Sugar Level
CAPN2	Caplain-2 Catalytic Subunit
CGM	Continuous Glucose Monitor
CNS	Central Nervous System
CRP	C-reactive Protein
CYP17A1	Cytochrome P450, family 17, subfamily A1
DEXA	Dual Xray Absorptiometry
DNA	Deoxyribose Nucleic Acid
FSH	Follicle Stimulating Hormone
GDM	Gestational Diabetes Mellitis
GIP	Glucose-Dependent Insulinotropic Peptide
GLP-1	Glucagon-Like Peptide-1
GLUT4	Glucose Transporter Type 4
GnRH	Gonadotropin Releasing Hormone
HbA1C	Hemoglobin A1C
HDL	High Density Lipoprotein
HIIT	High-Intensity Interval Training
HOMA-IR	Homeostatic Model assessment-IR
IC-PAMM	Insulin-Centric PCOS Analysis and Management Model
IGF-1	Insulin-like Growth Factor-1
IGT	Impaired Glucose Tolerance
IRS	Insulin Receptor Substrate
INSR	Insulin Receptor
IR	Insulin Resistance
LH	Luteinizing Hormone
ML	Machine Learning
MAPK	Mitogen-Activated Protein Kinase
MCP-1	Monocyte Chemoattractant Protein-1
MRI	Magnetic Resonance Imaging
NF-κB	Nuclear Factor Kappa B
NLR	Nucleotide-Binding Domain, Leucine-Rich Repeat Containing
NLRP3	NLR Family Pyrin Domain Containing 3
OGTT	Oral Glucose Tolerance Test
PCOS	Polycystic Ovary Syndrome
PI-3K	Phosphotidylinositol-3 Kinase
pmol/L	Picomole per Liter
QUICKI	Quantitative Insulin Sensitivity Check Index
ROS	Reactive Oxygen Species
RNA	Ribose Nucleic Acid
SGLT-2	Sodium glucose cotransporter-2
SHBG	Sex Hormone Binding Globulin
SNP	Single nuclear peptide
T2DM	Type 2 Diabetes Mellitis
TG	Triglyceride
VAI	Visceral Adiposity Index
WBC	White Blood Cell Count
